# Mutation or not, what directly establishes a neoplastic state, namely cellular immortality and autonomy, still remains unknown and should be prioritized in our research

**DOI:** 10.7150/jca.72628

**Published:** 2022-07-04

**Authors:** Shengming Zhu, Jiangang Wang, Lucas Zellmer, Ningzhi Xu, Mei Liu, Yun Hu, Hong Ma, Fei Deng, Wenxiu Yang, Dezhong Joshua Liao

**Affiliations:** 1 Department of Oncology, Taihe Hospital, Hubei University of Medicine, Shiyan 442000, Hubei Province, P.R. China.; 2 Department of Health Management Center, The Third Xiangya Hospital, Central South University, 138 Tong-Zi-Po Road, Changsha 410013, Hunan Province, P. R. China.; 3 Department of Medicine, Hennepin County Medical Center, 730 South 8th St., Minneapolis, MN 55415, USA.; 4 Laboratory of Cell and Molecular Biology & State Key Laboratory of Molecular Oncology, National Cancer Center/National Clinical Research Center for Cancer/Cancer Hospital, Chinese Academy of Medical Sciences and Peking Union Medical College, Beijing, China.; 5 Department of Pathology, School of Stomatology, Guizhou Medical University, 9 Beijing Road, Guiyang 550004, Guizhou Province, P.R. China.; 6 Department of Oral and Maxillofacial Surgery, School of Stomatology, Guizhou Medical University, 9 Beijing Road, Guiyang 550004, Guizhou Province, P.R. China.; 7 Department of Pathology, The Third Affiliated Hospital, Zunyi Medical University, Zunyi City 563000, Guizhou Province, P.R. China.; 8 Department of Pathology, The Affiliated Hospital, Guizhou Medical University, Guiyang 550004, Guizhou Province, P.R. China.; 9 Key Lab of Endemic and Ethnic Diseases of the Ministry of Education of China in Guizhou Medical University, Guiyang, Guizhou Province 550004, P. R. China.

**Keywords:** Epigenetic, Genetic, Neoplastic transformation, Stem cell, Tumor classification

## Abstract

Although the concept that cancer is caused by mutations has been widely accepted, there still are ample data deprecating it. For example, embryonic cells displaced in non-embryonic environments may develop to cancer, whereas cancer cells placed in embryonic environments may be reverted to phenotypic normal. Although many intracellular or extracellular aberrations are known to be able to initiate a lengthy tumorigenesis, the molecular or cellular alterations that directly establish a neoplastic state, namely cellular immortality and autonomy, still remain unknown. Hereditary traits are encoded not only by gene sequences but also by karyotype and DNA or chromosomal structures that may be altered via non-mutational mechanisms, such as post-translational modifications of nuclear proteins, to initiate tumorigenesis. However, the immortal and autonomous nature of neoplasms makes them “new” organisms, meaning that neoplasms should have mutations to distinguish themselves from their host patients in the genome. Neoplasms are malignant if they bear epigenetic or genetic alterations in mutator genes, i.e. the genes whose alterations accelerate other genes to mutate, whereas neoplasms are benign if their epigenetic or genetic aberrations occur only in non-mutator genes. Future mechanistic research should be focused on identifying the alterations that directly establish cellular immortality and autonomy. Benign tumors may have many fewer alterations and thus be much better models than cancers for such research. Future translational research should be aimed at identifying the cellular factors that control cancer cells' phenotypes and at establishing approaches of directing cancer cells towards differentiation, which should be a promising therapeutic tactic.

## Introduction

An adult person has about 1-3 × 10^13^ cells 1, 2, with 50-70 billion cells supplanted by the newly minted ones every day 3. However, there probably are not any two of these 10^13^ cells having exactly identical DNA sequences in the nuclear genome, which consists of 3.0-3.2 billion nucleotides in a diploid cell 4-6. This is because development from a fertilized egg to an adult person involves numerous rounds of cell division, and during these divisions a huge number of genetic alterations have occurred, leastways many changes in single nucleotides 7-11. These genetic alterations may occur via programs that had been evolutionarily entrenched in the genome, or may occur desultorily. An important piece of biological information that is less known to folks and even many biologists is that many genetic alterations, especially those occurring through genome-encoded programs, are beneficial 12-14 and required for the normal development and normal life of human beings 15, 16. For example, sister chromatid exchanges select good genes and pass them to daughter cells while purging detrimental alleles from the genome 17; meiosis creates haploidies so that the best parts of chromosomal DNA can be passed to the next generation 18-20, which, as already being noticed by Muller and others in the early 1930's 21, is an advantage of sexual reproduction. Moreover, establishment of acquired immunity involves genetic alterations to establish various strains of T and B lymphocytes that can respond to various pathogens 15, 22-24, and a large number of aneuploid hepatocytes are established in the liver to facilitate not only liver regeneration but also hepatic metabolism of various xenobiotics 25, 26. Different neurons in the same brain undergo different genetic alterations during the early ages of life to specify their functions 27-30, which may be a reason why some persons are smarter than others 31.

All genetic alterations are under close surveillance and strict control by the cells because their aberrances will likely lead to pathologies, typically cancer. Indeed, a 2013 Nature paper that analyzed mutations in over 7,000 cancers averred that “all cancers are caused by somatic mutation” 32, which projects a fact that neoplasms are widely perceived as diseases brought about by genetic aberrations 33. Actually, since 1956 mutations have been adopted by various nations' governments as a yardstick to assess cancer risk 34, 35, despite that soon afterwards it has been questioned 36-38. A poser our body faces is that many genetic alterations are beneficial and needed, and thus should be permitted and even encouraged, but this increases the chance for the bad alterations, which are usually dubbed as “mutations”, to mistakenly pass the surveillance and remain uncorrected. If evolution had not programmed genetic alterations in our genome and had not permitted any genetic change to happen, we would have many fewer worries about various repercussions (such as cancer), but, meanwhile, we would not have an exquisite brain with so diversified neurons and would not be able to live a happy life in the Mother Nature that is fraught with pathogens and harmful materials. For instance, gene fusions often occur in leukemia and lymphoma, which in our opinion is related to the fact that their normal parental cells perform genetic modifications to evolve themselves into functional immune cells. Unfortunately, human beings face too many “buts”.

## “Cancer is caused by mutation” has become a “cancer 101”

Classically, genetic alterations are stratified into three different levels, i.e., the cellular level shown as changes in the chromosome number, the chromosomal level manifested as alterations in the chromosomal structure such as a deletion or an amplification of a DNA region, and the DNA sequence level exhibited as changes in single nucleotides. Changes in the chromosome number, either hypoploidy, hyperploidy, or aneuploidy, can be regarded as an enlarged version of deletion or amplification of DNA sequences. For simplicity, these three levels of alterations are collectively coined herein as “mutation”, although in many publications “mutation” is only used to indicate single nucleotide changes. A huge number of hereditary diseases, such as Down syndrome (trisomy 21 syndrome), have been causally linked to alterations at one or more of these levels, making it clear that hereditary traits are encoded not only by DNA sequences (i.e. genes) but also by chromosomal structures and chromosome number. Heng et al have further pointed out that, while genes encode “parts” of inheritance, chromosomal structure and karyotype encode “system inheritance”, which usually is 'fuzzy” and does not follow Mendelian genetic law 39-42.

Ever since an abnormal number of chromosomes was observed in cancer cells in 1875 43-45 and had later, during the turn from the 19^th^ to 20^th^ centuries, been propounded by Hansemann and Boveri as a cancer cause 44, 46-50, the idea that formation and progression of cancer are attributed to mutations has gradually become the orthodox doctrine of carcinogenesis research 51-57. With accumulation of clinical and experimental evidence, Nordling formally proffered the first mutation theory of carcinogenesis in 1952 (published in 1953) 58 and, since then, there have been many discourses on the causal relations of various types of mutations to tumorigenesis or carcinogenesis, which are herein referred collectively to as “mutation theory”. The Science issue of November 22, 1991 was devoted to this mutation concept 59, making it even more popular in the cancer research fraternity in recent decades. In 1976 Nowell proffered a stepwise concept of mutation-caused carcinogenesis 60, 61: A cell's genome somehow goes awry and becomes unstable, thus randomly resulting in more and more mutations when cells replicate. These mutations serve as raw materials for the cells to select the beneficial ones to become fitter mutant clones 62, 63. In most cases cancer cells in the same patient are greatly heterogeneous in their morphology and comportment, which has also been imputed to their accrual of various mutations 62, 63. In this “bi-phase” process, i.e. random mutations followed by clonal selection and expansion of the fitter mutant(s), genetic instability as the initial cause often results in chaotic karyotypes, at least in the cells that bear mutations in the p53 gene 40-42, 64-66.

## Changes at some higher levels of genetic control may also initiate tumor formation

**Figure 1:**
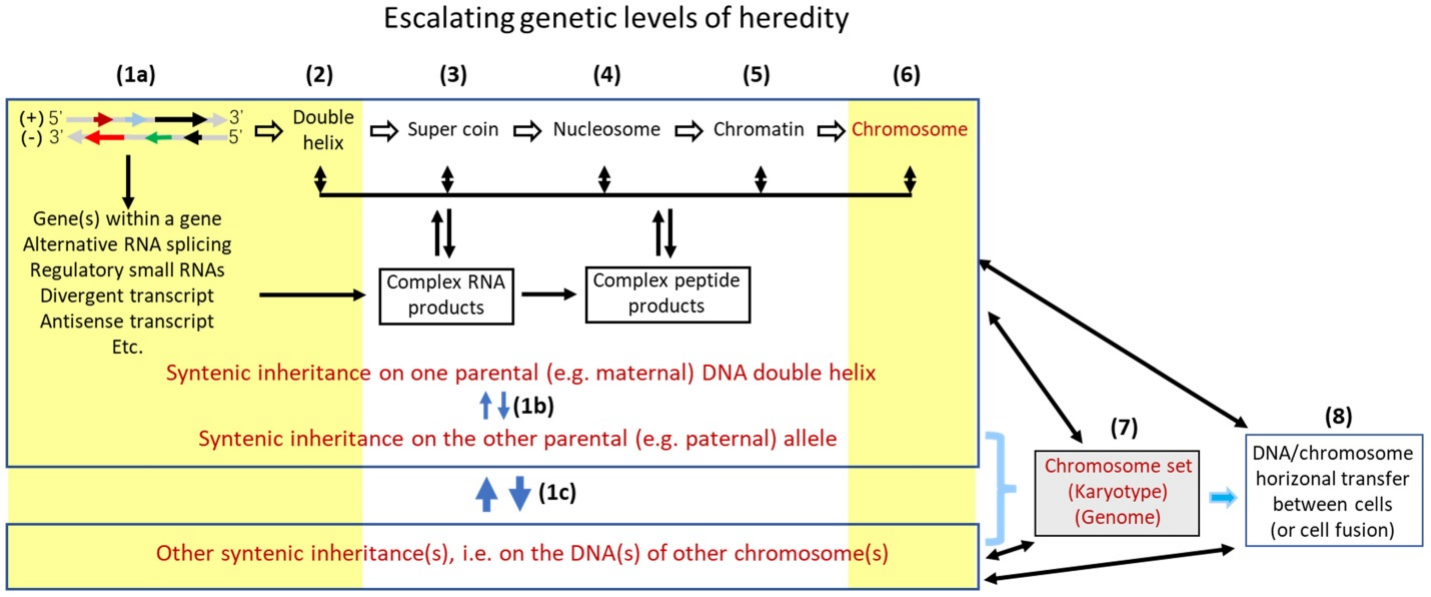
** An oversimplified representation of multilevel structures of genomic control of inheritance.** The lowest level (level 1a) is the two genomic DNA sequences that harbor genes, i.e. the plus and minus strands of DNA. On the (-) strand, the gene indicated by the short black arrow is embedded in the long-black-arrow gene on the (+) strand 68, while the gene indicated by the green arrow partly overlaps, reverse-complementarily, with the long-black-arrow gene. Another gene on the (-) strand (the long red arrow) not only has an antisense RNA (the short red arrow) but also has a divergent transcript (the short blue arrow), both on the (+) strand. The many introns of the transcripts from this genomic locus are also processed to different small regulatory RNAs (e.g. siRNA and microRNA) that are not shown in the figure to avoid overwhelming it. These genes may interact with their counterpart allele on the other parental chromosome, which constitutes another sub genetic level (1b). Furthermore, one gene on one chromosome may collaborate with another gene on another chromosome, as having been shown by many bitransgenic or double knockout models of animals, which also constitutes an additional sub level (1c). Changes at these three sub levels that involve DNA sequences have been extensively studied for their roles in tumorigenesis (denoted with a yellow-shaded area). However, several higher levels (levels 2, 3, 4 and 5) of genetic controls, i.e. the levels at the formation of double helix, super coin, nucleosome, and chromatin, each of which encodes a set of hereditary traits that is more complex than the set controlled at a lower level, have been much understudied for their effects of alterations on tumorigenesis in part due to technical constraints. Fortunately, alterations at the two higher levels (levels 6 and 7) that deal with chromosomal structure and number, such as chromosomal translocation, aneuploidy, etc., have received relatively-more extensive studies for their contribution to tumorigenesis (denoted with a yellow-shaded area), although it seems that these lines of studies have gradually faded in the past 20 years or so. Studies on the level 8, i.e. the roles of DNA horizonal transfer (including fusion of two cells' genomes) in tumorigenesis have, in general, been understudied as well.

In our opinion, there are at least eight tiers of genetic control that escalate in the complexity and the influence on inheritance, with the DNA sequence as the bottom level (Fig. 1). Each higher tier encodes a set of hereditary traits that is more complex and probably “fuzzier”, compared to the set controlled by a lower tier. Karyotype studies, which analyze the structure and number of chromosomes and were popular in cancer research during the 1960's-2000's, had provided a profusion of data for the establishment of mutation theories 67, although, as pointed out by Heng et al. 41, 42, it is pity that this line of research has seemed to fade out in the past 20 years or so. The swift promulgation of DNA sequencing technology in recent decades has provided deep mechanistic insights into the effects of altered DNA sequences on tumor formation. However, in our opinion the intermediate levels between the gene sequences and the chromosomal structures shown in Figure 1 remain much understudied for their effects of alterations on tumor formation. Abnormalities at these intermediate tiers of genetic control, e.g. changes in nucleosomes, may have more systemic and thus more complicated contributions to formation of tumors, especially the malignant ones, compared with altered DNA sequences. Besides certain types of DNA mutations such as gene deletion or amplification, certain aberrant post-translational modifications of nuclear proteins, such as aberrant phosphorylations of histones, may alter some of these intermediate genomic structures, in turn altering hereditary traits or predisposing the afflicted cells to neoplastic transformation.

## There are many theories that dissent from the mutation one

While there are voluminous data undergirding the aforementioned mutation theory, there also are profuse biological phenomena and experimental data suggesting that mutations may not necessarily be required for cancer formation 56, 69-77 and even for the heterogeneity of cancer cells 78, as first broached by Rous in 1947 79. Actually, this constellation of data has led to formation of many theories and models of tumorigenesis or carcinogenesis that are collectively referred to as “non-mutation theories” herein 56, 80-94. These divergent non-mutation theories include (but are not limited to) the “tissue organization field” theory (TOFT) 56, 91, 95-99, the “dynamic developmental disorder” theory 53, 100, the “population dynamics of cancer” theory 74, 101, the “dynamical non-equilibrium systems” theory 102-104, the “embryonic morphogenetic field” theory 94, the self-disorganization theory 105, 106, the eco-evolution, speciation, or atavism theory 107-112, the systemic-evolutionary theory 113, 114, the “cell reversal” theory 115, the karyotypic theory 116, the chaos theory 117, the pericyte hypothesis 118, the “activity paradigm” theory 119, etc. These deprecatory theories usually overlap with, or are complementary to, one another 120. Many of them do not completely foreclose the mutation theory 121 but, instead, attempt to integrate with it to form a conflated theory, such as the “emergence framework of carcinogenesis” theory 122-126, the molecular theory 121, etc. The data as the raison d'être of these deprecatory theories are synopsized below, with the relevant history provided to our best knowledge and with our somewhat provocative perspectives elaborated. The terms “tumorigenesis” and “carcinogenesis” are used in different places in part because the described process sometimes implicates formation of benign tumors.

### Dissenting evidence 1: Some biological phenomena are paradoxical to the mutation theory

**Figure 2:**
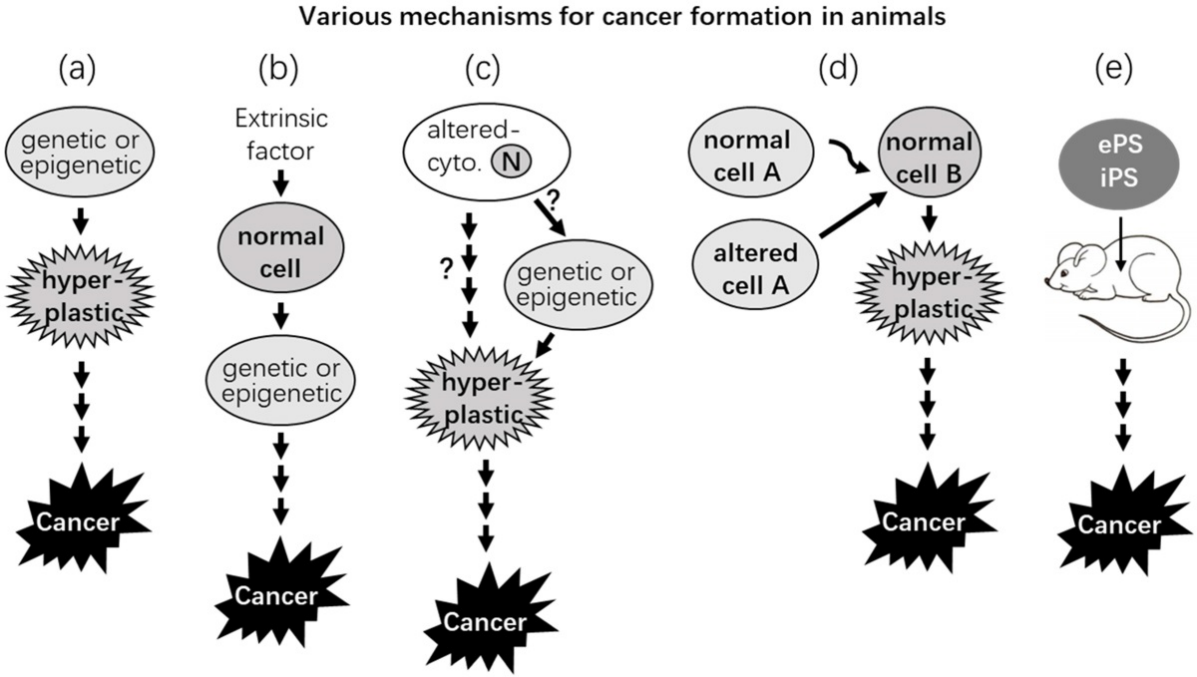
** Almost any aberration inside or outside a cell, mutational or not, may initiate cancer formation. (a)** A primary cell may bear certain epigenetic or genetic alteration(s), such as one inherited from a parent, that enable the cell to proliferate and form a hyperplastic lesion while gradually becoming cancerous. **(b)** Certain extrinsic (extracellular) factors, such as radiation, a chemical, a virus, or an abnormal endocrine or paracrine signal, can cause genetic or epigenetic change(s) in the nucleus of a cell, either directly or via altering certain cytoplasmic factor(s), and make the cell cancerous, as in (a). **(c)** Hypothetically (the question marks), certain factors in the cytoplasm may become abnormal, due to such as an unhealthy lifestyle or aging, which renders the cell hyperplastic either directly or by causing genetic or epigenetic alteration(s) in the nucleus (N), driving evolution of the cell to a cancer. **(d)** Some cytoplasmic or nuclear alterations of some cell(s) (such as stromal cells) may alter their communications and interactions with other (such as epithelial) cell(s). The alterations may direct evolution of the latter cell(s) to cancers while the former cells remain phenotypically normal. **(e)** A normal embryonic or induced pluripotent stem cell (ePS or iPS) may develop into a cancer at an ectopic (i.e., extrauterine) place in adult animals.

Species of larger animals are supposed to have higher cancer incidences than the smaller ones, because a larger body has experienced more rounds of cell replication and thus has encountered more chances for spontaneous mutations to occur (Fig. 2). However, this is not shown in reality, as was pointed out by Peto in 1975 and thus is dubbed as Peto's paradox 127-131. Usually, cell proliferation is quicker and more robust during the early part of life, and thus mutations and cancers should occur more often in the young if mutations are directly responsible for cancer formation. However, cancers occur more often in the elderly 132, 133, although very old individuals often have decreased cancer incidences 134-137. Of course, there are pediatric cancers, which are likely initiated during the embryonic stage 138, 139 and occur via different mechanisms from those for the sporadic cancers in adults 140. A related perplexity is called “the proliferation paradox” 141: Some cell types that have the fastest turnover in the human body have the lowest cancer incidences 141, epitomized by the epithelial cancers in the hair follicles and the small intestine. Epithelial cells of the hair follicles grow so fast that men need to cut their hair once a month, but these cells rarely develop to cancer 142, 143. Small intestine makes up 75% of the length and 90% of the mucosal surface area of the digestive tract 144, and the lifespan of its mucosal cells is so short that the cells are supplanted by the newly minted ones every 3-4 days 3, 145. However, the incidences of epithelial cancer in the small intestine, especially in the jejunum and ileum, are extremely low 144, 146-148, in stark contrast to the cancer incidence in the colon and rectum that are much shorter in length with their liner epithelia having a much longer (5-21 days) lifespan 3, 145.

There are many genotoxic agents that are not carcinogens 149, whereas a large percentage of known chemical carcinogens are non-genotoxic, such as chloroform and p-dichlorobenzene 150, 151. Endogenous hormones can beget benign or malignant tumors when they are present in an aberrant amount, which can be achieved using simple surgeries such as partial thyroidectomy 138, 152, 153, gonadectomy, and transplantation of gonads to an ectopic body site (such as to the spleen) 154-157, as we have reviewed before 138, 158-160. Obviously, endogenous hormones and simple surgeries cannot be considered mutagenic. There are too many other factors that are not mutagenic but can increase risk for cancer, such as obesity and certain unhealthy lifestyles.

There are some cancers in which no recurrent mutations could be identified 161, 162, and there has not been any proven set of mutations known to transform a normal cell to a cancerous one 163. More bewilderingly, there are some oncogenic driver mutations appearing in benign diseases at a high frequency, sometimes even much higher than in malignant tumors 164-166. There also are cancer-driver mutations that are found in normal cells or culminate in only clonal proliferation of normal cells, but not cancers 132, 133, 167, 168. A conjecture on these observations is that, besides causing neoplastic transformation, these mutations can also improve fitness of relatively old cells and thus extend their life span; therefore, there is no need for the mutations to drive these fitter cells to a neoplastic state 132, 165, 168. All of the observations enumerated above do not seem to be consonant with the mutation theory, although there may be other explanations.

### Dissenting evidence 2: Altered extracellular milieu may initiate carcinogenesis

There have been several lines of experimental data intimating that abnormal extracellular signals from the matrix or from other cells may initiate neoplastic transformation 169, 170, which occurs even in evolutionarily very low animals like metazoan Hydra 171, 172. Actually, there is a theory opining that cancer is a problem in intercellular communication 173. One line of advocating data is derived from many animal studies showing that implantation of various foreign bodies can cause tumors, mainly sarcomas 174-177, which was first reported by Turner in 1941 who fortuitously found that subcutaneous implantation of Bakelite disks in the rat caused sarcoma at the site of implantation 178. Implantation or chronic injections into animals' peritoneal cavity of different non-mutagenic materials, such as solid plastics, mineral oil, and certain immunological adjuvants, can induce plasmacytomas, which has been reviewed by Potter decades ago 179-181. These implanted materials fall into various categories, including metal, plastic, polymers, millipore filters, nitrocellulose, etc., and are insoluble and not toxic 182-188. Moreover, the carcinogenesis does not seem to correlate with the amount (dose) of the implanted materials, but rather is related to their physical shape or surface 176, 188-190. Therefore, the carcinogenesis does not seem to occur via mutations caused by the intake of the materials into the cells but rather occurs due to disturbances to the extracellular milieu (Scenarios b and d in Fig. 2). It needs to be emphasized that such foreign-body-caused carcinogenesis has its human relevance. For example, there have been over 800 cases of “breast implant-associated large cell lymphoma” reported in the literature 191. Moreover, soft tissue malignancies caused by foreign bodies derived from shotgun blasts have also been reported 192. Relevant mechanistic studies in the past 80 years suggest that the carcinogenesis is likely to be ascribed to the chronic inflammation ignited by the implanted materials, such as the involvement of macrophages, plasma cells, and other inflammatory cells as well as various cytokines and other factors released by these cells 174, 189, 193. Actually, Miller has already shown in 1931 that injections of tuberculo-proteins into the peritoneal cavity of rabbits can induce nodules in the omentum that contain “undifferentiated cells” 194, which are neoplastic in pathology term. In our opinion 138, this carcinogenesis is likely elicited via chronic inflammation, but not mutations, caused by the bacterial proteins, and supports Rudolf Virchow's theory that cancer results from chronic irritation 195-198, mainly inflammation 199-201.

Another line of espousing data is derived from experiments of transplantation of tumor tissues into normal animals 202-204. According to Staab 205 and Goldenberg 206, during 1905-1907 Ehrlich, Apolant, Loeb, Bashford, and a few others had reported in German language that transplantation of mouse mammary carcinoma into other mice could culminate with sarcomatous transformation of the recipient's stromal cells. Actually, in the 1902 Loeb had already noticed that the regrowing tumors in the recipient animals were sarcomas although the original tumors implanted were carcinomas 207, which suggests a possibility that the tumors occurring in the recipient animals may not really be a regrowth of the grafted tumor but may rather be a new tumor derived from a different cell lineage. Unfortunately, the importance of these earlier observations had been ignored for decades, and it was only in the 1970's was it shown in a series of studies that inoculation of surgically-removed human cancer tissues into immunodeficient mice, rats or hamsters, followed by poly-passages of the transplanted tumor tissues from one animal to another, can cause sarcomatous transformation of the rodents' stromal cells within the grafted human tumor tissues 205, 206, 208-213. The detailed mechanisms underlying this horizontal transformation of malignancy remain nebulous even now. Possible explanations include that certain transforming-genes have been horizontally transferred from the primary tumor cells to some recipients' cells 213-219, that spontaneous mutations have occurred in some recipients' cells, and that immunodeficient animals have already borne certain mutations that drive malignant transformation of their stromal cells. However, in our musing these possibilities are improbable, partly because inoculation of well-established cancer cell lines 206, 220, such as several subclones of Hela cells 221-223, into nude mice culminate only in metaplasia, typically bone or cartilage formation, and not neoplastic transformation, of the recipients' cells. These discrepancies between inoculation of a cancer tissue and inoculation of a single cancer cell line intimate that heterogeneous populations of cancer cells and/or connective tissue components in the donor cancer may contribute to the neoplastic transformation of the recipients' stromal cells.

Ever since 1951, another set of tissue graft studies has also reached the conclusion described above: Billingham et al repeatedly painted some areas of mouse skin with 20-methylcholanthrene, a chemical carcinogen, and then removed the epidermis and implanted it onto an untreated area of dermis with the epidermis pre-removed 224. Unlike other painted areas that developed many tumors, no tumors developed at the transplanted epidermis. Conversely, if implanting a pad of epidermis from an untreated area to a treated area with the original epidermis pre-removed, tumors would develop at the untreated epidermis. Obviously, the tumorigenesis in the graft of untreated epidermis is begotten by the deeper, carcinogen-treated tissues 203, 224, 225. Similarly, non-tumorigenic COMMA-D cells inoculated into a mouse mammary fat-pad that was previously irradiated and cleared of epithelial cells developed to cancer 226-229. Normal rat mammary epithelial cells inoculated into a mammary fat-pad of a rat that was previously treated with the chemical carcinogen N-nitrosomethylurea developed to cancers as well 56, 92, 98, 230. In these experiments, mutations may still contribute to the tumorigenesis, but in such a way that a cell or cells bear mutation(s) and therefore keep providing a disturbing signal to other cells, eventually making the latter neoplastic (Fig. 2). Supporting this conjecture, normal ovaries grafted into the spleen 154, 156, 231 or tubal eggs grafted into the testis 232 have since the 1940s been shown to develop tumors. The ectopic site, i.e., the spleen or the testis, should not be mutagenic, but it provides a long-lasting disturbance to the grafted cells. Mention should be made of similar results from many unethical (likely criminal by today's law) studies involving inoculation of cells directly into human bodies performed mainly during the 1940's-1960's 233-237. For instance, it had already been reported in Science in 1956 that subcutaneous inoculations of cultured human epithelial cell lines into forearms of human “volunteers” could lead to tumor formation, although the tumors eventually regressed 238, 239, likely due to immune clearance by the recipients 240. The normal recipients' forearms are not mutagenic but can still neoplastically transform the inoculated epithelial cells.

### Dissenting evidence 3: Primary rodent cells readily immortalize themselves *in vitro*

Normal somatic cells have allegiance to the host's body and are mortal, as they have lifespans. A neoplastic state of a cell means that the cell has lost its allegiance to the host's body 111, 138, 139, 241, 242. In other words, a neoplastic cell, benign or malignant, has become autonomous and maintains itself as a unicellular organism by interminable symmetric division, namely becoming immortal 243, 244, just like a bacterial cell that keeps symmetrical division to maintain its strain 111, 138, 241. Unfortunately, many studies on neoplasms do not stick with this “immortality and autonomy” definition of neoplasia 138 but describe tumors in various ways, as summarized by Gatenby et al 245 and Soto et al 246. Some lesions that are described as benign tumors in pathology textbooks are not actually neoplastic because they are not immortal. For instance, many osteochondromas cease growth and even diminish after skeletal maturity 247-250, and thus should be considered as developmental malformations, but not neoplasms. A caveat is that in reality, every sizable tumor mass has a sheer number of neoplastic cells that are either dying or already dead (mortal) or have already committed to mortality, i.e., have lost the ability of interminable self-renewal, due to variegated reasons such as desultory development of lethal mutations or insufficient supply of oxygen or nutrients. This reality should not disqualify immortality and autonomy as the cannons for neoplasia. Unfortunately, as we have pointed out and discoursed recently 138, it has led to a wide misconception in cancer research that only a tiny number of cells in a cancer mass encompass the self-renewing ability and these cells should thus be specifically classified as “cancer stem cells” to be distinguishable from the remaining vast majority of cancer cells.

The neoplastic nature of cells *in vitro* is usually referred to as “neoplastic transformation” or just “transformation”, which is equivocal as it does not clearly announce whether the “transformed” cells are immortal and/or autonomous 251. Cells in culture dishes can only be evaluated for their immortality with their ability to be passaged endlessly, whereas their autonomy cannot be assessed, unfortunately, because no allegiance to the host animals is involved 111, 138, 241. Actually, even “unlimited passage” is difficult to assess as it requires continuous passage for a long period of time, and currently we still lack a feasible approach to determine the turning-point from mortality to immortality of cells in culture.

Carrel and his associates Burrows and Ebeling had since the 1910's presented a series of publications claiming their successful *in vitro* immortalization of chick embryonic fibroblasts by continuing adding chick embryo extracts into the culture of chick embryonic heart tissue, although many contemporaries questioned this world's first success in transforming cells *in vitro* 252-255. Nevertheless, there have since 1940's been many studies showing that *in vitro* culture can easily transform primary cells of small rodent origins 71, especially the hamster and mouse 256-260, as the cultured cells can form tumors when injected into syngeneic animals. A so-called “3T3 protocol”, namely transferring 3 x 10^3^ cells from one flask to another every 3 days, had been established in the 1960's as an effective procedure to immortalize primary mouse fibroblasts, especially those from early embryos 261-263. Rodent epithelial cells can easily transform themselves *in vitro* as well, which has been postulated to be due in part to the disruption of their interactions and communications with stromal cells. Moreover, isolation of epithelial cells detaches them from the basement membrane, which has been known for decades to facilitate immortalization 106. In general, immortalization or neoplastic transformation of primary cells is much more efficient, once estimated to be 10^10^ times better 264, *in vitro* than *in vivo* 105, 106. Treatment with various non-mutagenic agents can facilitate *in vitro* immortalization and neoplastic transformation. For example, a low dose of hydrogen peroxide can cause a transformation 265.

The cell culture situations enumerated above are stressful to the cells, which is likely to cause chaos of the karyotype, especially when the p53 gene is also mutated, as observed by Heng et al. 40-42. However, in our opinion the stress itself may not be mutagenic; epigenetic alterations may more likely be the initial events occurring in the cultured cells whereas genetic changes, if they occur, may be the secondary and spontaneous ones. Indeed, it has been known that many spontaneously-established cell lines show deletion in the INK4a/ARF locus 266-268, besides methylation of the p16 gene within this locus 269, 270.

### Dissenting evidence 4: Mutations rarely transform cells in animals

Although some inherited mutations are associated with higher tumor incidences 271-273, one particular inherited mutation culminates with only one or several tumor masses in only one or several cell or tissue types. For instance, an inherited mutation in the Rb gene may cause retinoblastoma, but often only one tumor is developed, although not only all of the retinal cells in both eyes but also all of the cells in the patient's body bear the mutation 274. This means that there are inherent factors in the body that prevent the vast majority of the incriminated cells from the mutation-initiated carcinogenesis 132. This phenomenon can also be discerned in most animal models of solid tumors with genetically manipulated mice, as we have repeatedly pointed out before 6, 138, 275: In these models, although all animals may develop the anticipated tumor, usually each animal develops only one or several overt tumors during the life span, despite that the target organ of the animal, such as the liver or the mammary glands, have trillions of cells that bear the same genetic modification, as having been noticed decades ago 276, 277. For instance, only 4 or 5 islets in the pancreas of SV40-LT transgenic mice develop β-cell tumors 278, and only 1 among 10 mammary glands in c-myc transgenic mice develops a tumor 279, 280, although we did occasionally find two or three mammary tumors in a mouse in our lab (empirical experience of DJ Liao). To our knowledge, these two transgenic lines are already ones of the best models of carcinogenesis as they produce the highest tumor incidences. The fact that only one to several out of trillions of targeted cells in the same animal are transformed early enough for the cells to develop to overt tumors signifies that the genetic manipulation as an artificial mutation has negligible efficacy in neoplastic transformation 6, 138, 275. The discrepancy between 100% tumor penetrance at the animal level and the negligible transformation efficacy at the cellular level is reminiscent of the situation in the human being that “cancer is so common a disease yet so rare at a cellular level”, as pointed out by Ferrell Jr et al 281. Indeed, one of five people will likely develop cancer in his/her lifespan 142, 143, which is horrible. However, since one person has 1-3 x 10^13^ cells with 50-70 million cells supplanted by newly minted ones every day 2, 3, this still means that the rate of cellular neoplastic transformation is negligibly low, much lower than 1/10^13^.

### Dissenting evidence 5: Mutations are not required for showing neoplastic properties

Benign neoplasms are already immortal and autonomous, and malignant neoplasms have three additional features, i.e., 1) encroachment into their normal adjacent tissue, which can be considered as local metastases, 2) consumption of their normal surrounding tissue, which can be regarded as a cannibalism at the cellular level 282-286, and 3) metastasis to distant body site(s). The mutation theory contends that a normal cell develops mutation(s) to evolve to a neoplasm, and then develop more mutations to acquire the three malignant features. However, none of the five neoplastic properties are unique to malignant cells, and not even to benign cells, as these cellular properties are developed along with evolution from prokaryotic to eukaryotic and then to multicellular organisms. In other words, the genomes of animals (including the human being) encode these cellular properties and thus do not need mutations for their occurrence 62, 287, 288: First, a normal human body consists of not only somatic cells, which are mortal and may undergo symmetric division, but also germline cells that are immortal and undergo asymmetric division 289, 290. Actually, there are some plants and animals that are immortal as well 290, 291. Therefore, immortality has been evolutionarily built within, or encoded by, our genomes although normally the program is derelict in somatic cells. An intriguing but unsolved question is how autonomy is related to immortality and whether it is also evolutionarily built within the genomes of multicellular organisms. In our cogitation, immortality and autonomy are the two sides of the same coin for neoplastic cells, meaning that they are controlled by the same factors that are currently unknown to us 138, as there is no evidence showing extrication of immortality from autonomy in human tumors. Second, invasion is an evolutionarily-developed cellular comportment seen widely in normal cells of animals and plants 292. For instance, normal trophoblasts are highly invasive 293, 294 and can make inroads into the uterine wall to establish gestation and may even encroach into blood vessels and home in on the lungs of the mother and many organs of the newborn 295. Third, macrophages and even some other cell types like epithelia can engulf other cells and materials in their surroundings; osteoclasts function to eat up bone tissue 296, 297. Fourth, many bone-marrow-derived or thymus-derived cells can enter, i.e., “metastasize”, into the blood or lymphatic circulation and home in on almost anywhere in the body. Probably because of this property, in all pathology textbooks neoplasms of the bone-marrow and lymphatic origins are all classified as malignancy without exception. Fortunately, this property seems to have its benefits: Because these neoplasms, which usually are liquid cancers, do not need to experience additional cellular or molecular changes to be metastatic, many of them have fewer alterations and are easier to cure, compared to many solid tumors 298. Actually, during embryonic development many cells migrate, with an instructive embodiment already described by Markert in 1968: “…melanoblasts originating in the neural crest migrate through many tissues of the body before reaching the terminal locations in which they complete their differentiation into nondividing, nonmigrating melanocytes” 288. As we have described before 111, 138, 139 and in this essay, carcinogenesis is an atavistic process and cancer cells resemble embryonic cells in morphology and comportment, and metastasis of cancer cells may be considered as showing behavior of embryonic cells. Actually, for this reason pathologists use embryological terms, such as “undifferentiated”, “poorly differentiated”, “differentiated”, etc., to describe neoplasia 299.

According to the systemic-evolution theory of Mazzocca et al 113, 114, 300-302, other neoplastic properties, such as fermentative glycolysis, are also entrenched in the genomes of eukaryotes that evolve from fusion of two different types of prokaryotes, with one of the erstwhile types now being represented by the nucleus and the other being represented by the mitochondria. This explains why cancer cells sometimes, but not always, manifest fermentative glycolysis. Many intracellular or extracellular disturbances, including epigenetic perturbations, may reactivate some of these derelict programs, making the cell stay at or return to an embryonic (or stem) stage to become neoplastic 303-305. From a vantage point of logic, even very egregious cancer properties do not need to be derived from mutations 56, because they have already been entrenched in the normal genome, mostly in the genomic DNA and, probably, mildly in the mitochondrial DNA. Of course, some mutations may bestow these properties upon cells while some other mutations (such as a deletion) may make them disappear. This point of view is not just a logical inference but has actually been buttressed by many experimental data, as have been summarized by Pierce in 1983 306.

### Dissenting evidence 6: Pluripotent stem cells may develop into cancer at extrauterine sites in adult animals

According to Needham 307, Belogolowy showed in 1918 that morulae and blastulae of anuran amphibia implanted into tissues or body-cavities of adult frogs developed into “round-celled sarcoma” that penetrated into the surrounding tissue and metastasized to the liver and lungs. Also according to Needham 307, Skubiszewski reported in 1926 that injection of chick embryonic tissue into chicken muscle or other tissues produced similar “round celled sarcoma”. In the 1930s, both Needham and Thomas observed oocyte-caused tumors in adult worms 307. Witschi in the 1930s showed that if frog eggs were kept for a prolonged period of time before fertilization by sperms, which is referred to as over-ripeness, the eggs would produce teratomas or teratocarcinomas 307-309. In 1960's, Steven et al showed that, when germinal stem cells from early embryos of male mice of the 129-strain were transplanted into testicles of adult mice, the cells developed into teratomas or teratocarcinomas (Fig. 2) 232, 310, 311. As reviewed by many pundits 312-327, many other researchers have later confirmed that early embryonic pluripotent stem (ePS) cells, including those of human origin 316, placed into several extrauterine sites of adult animals can indeed develop into teratomas or teratocarcinomas 315, 328-332. Sobis et al 333-340 and Hirai et al. 341 have also shown that displacement of yolk sac cells in fetectomized placenta induce teratomas and teratocarcinomas in small rodents.

A host of studies in the past decade or so have confirmed and extended the earlier findings mentioned above on the development of teratomas or teratocarcinomas from induced pluripotent stem (iPS) cells 312, 342-347. It is now clear that either ePS or iPS cells may develop into teratomas and even teratocarcinomas if the cells are placed ectopically, i.e., at an extrauterine site of adult animals (Fig. 2). The tumorigenic mechanisms, according to Rose's work in 1955 with embryonic and adult frogs, may involve inhibition of differentiation of the pluripotent cells by the adult tissue matrix 348. As extrauterine sites in animals should not be mutagenic, this tumorigenesis or carcinogenesis may not involve mutations. Moreover, the tumor formation can be greatly minimized or prevented by various manipulations 312, 344, 345, 347, 349, which also favors the perception that the tumorigenesis is mainly precipitated by the non-mutagenic microenvironment.

### Dissenting evidence 7: Embryonic environment may revert cancer cells back to normal

Mutations are in general considered irreversible 92, although sometimes polyploidy of cancer cells may be reversible 350, 351 and in some rare cases single nucleotide mutations may mutate back to the wild type 22, which had been described already in 1940's 352 and coined as “reverse mutation” or “back mutation” 353-357. The irreversibility of mutations dovetails with the fact that human cancers rarely regress spontaneously. Of course, there are some rare cancer subtypes showing high frequencies of spontaneous regression with unclear reasons, such as the stage IV-S of neuroblastoma 358-360, some indolent histologic subtypes of non-Hodgkin's lymphoma 361, and some subtypes of cutaneous malignant melanoma 362, 363. A caveat is that many precursor lesions in animal models of carcinogenesis 138 and certain outgrowing lesions of humans 105, 106 can regress via apoptosis because these lesions are still mortal and have not yet become authentic neoplasms 138.

**Figure 3:**
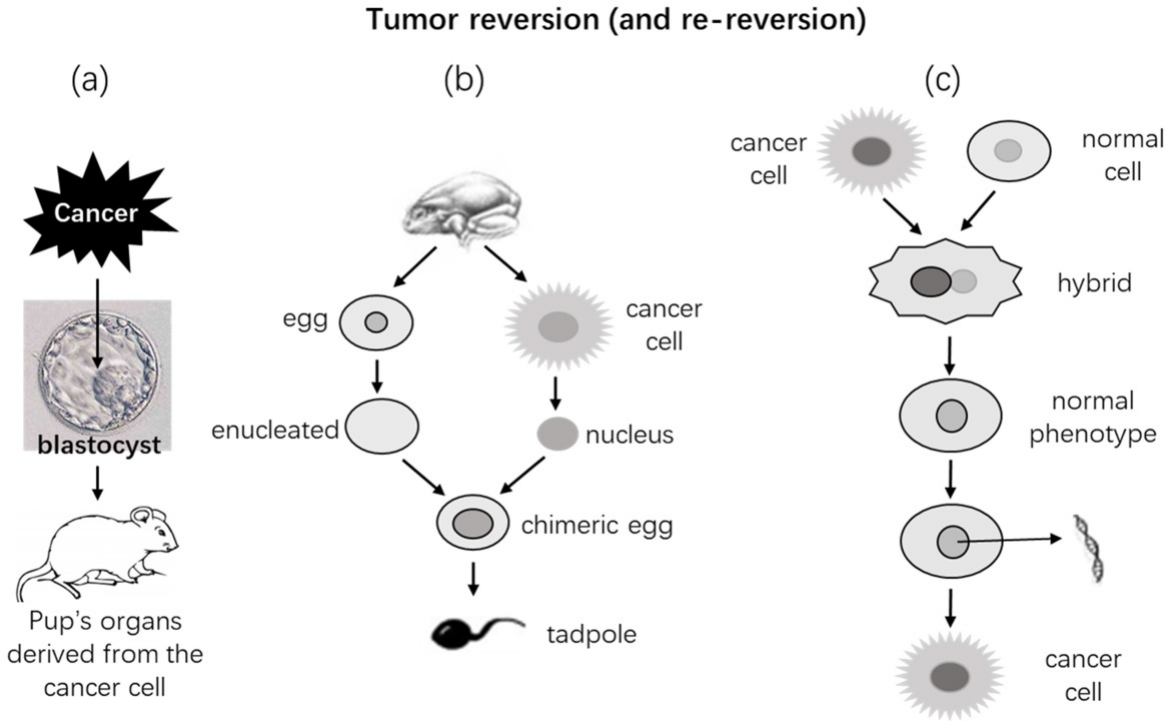
** Several modes of reverting cancer cells back to normal. (a)** If a mouse cancer cell is injected into a mouse blastocyst, it would develop together with the embryonic cells into an embryo and then to a fetus. **(b)** If a nucleus isolated from a Lucké cancer cell of frog origin is injected into a denucleated frog egg, the chimeric egg can hatch a normal tadpole, showing that the normal cytoplasm of the egg overrides the cancerous nucleus in controlling the cellular and organic phenotypes. **(c)** Fusion of a normal cell with a cancer cell may make the hybrid phenotypically normal. However, removal of certain chromosome(s) from the hybrid that has already been normalized may revert it back to the cancer phenotype again 415-424.

While ePS cells may develop into cancer in extrauterine matrices, ever since 1907 364, 365 a myriad of animal studies have also shown that cancer cells may be reverted back to normal in an embryonic environment as well (Fig. 3). When teratocarcinoma cells were injected into mouse blastocysts, the cells became incorporated into the developing embryos; organs and tissues of the adult mice developed from such embryos consisted of cells from both the normal blastocyst and the cancer (Fig. 3) 94, 306, 366-376. Actually, a similar observation was already made in 1907 by Askanazy 377 who, according to Telerman 378, showed that ovarian teratoma cells could differentiate to normal tissues that contained embryonic germinal layers. Since the late 1950s, Pierce and his colleagues have further shown that a single cell of teratocarcinoma or some other cancer types can develop to the three major germ-cell layers of embryos 326, 379-385. After being frozen-and-thawed *in vitro* for many times, cells of teratocarcinomas that were derived from mouse embryonic cells could still be made to develop to gametes, and the oocytes or sperms could generate normal progeny 386, 387. Cells of other tumor types such as leukemia and neuroblastoma have been shown to be regulated by certain embryonic fields as well 306, 388, 389. However, it seems that the embryonic environment's control over malignant phenotypes has its specificity, since the blastocyst fails to control certain leukemia and sarcoma cells 376 and only tumor cell types with a normal cellular counterpart in the blastocyst could be well controlled 306. More interestingly, treatment with Zebrafish embryo extracts, alone or in combination with certain chemo-drugs, has been shown to have therapeutic effects on liver cancer and breast cancer both in the lab and in clinical trials 390-394. The microenvironment of mammary tissue can direct differentiation of breast cancer cells as well as normal cells of certain tissue origins (such as testes and nerves) 395, 396. A regenerating mammary gland can also provide a special milieu in which human breast cancer cells can be reverted to mammary epithelial cells 397, 398. These mammary-gland-related data are somewhat related to the effects of embryonic environments, because the mammary gland is special as it starts to develop only at the pubertal age and becomes mature only after parturition.

It had been shown in the 1960's that if nuclei isolated from the Lucké renal cancer cells of frog origin 399-402 were injected into enucleated frog eggs, the chimeric eggs could hatch phenotypically normal tadpoles (Fig. 2) 403-413. Further transplantation of tissues from these tadpoles into normal recipients produced phenotypically normal tissues as well 407. Similarly, if nuclei isolated from cells of mouse medulloblastoma are injected into enucleated mouse oocytes, the chimeric eggs can develop to embryos in recipient female mice, and the embryos can survive for 8.5 days of the embryonic stage with various normal embryonic tissues and without showing any neoplastic features 414. These observations further extend the aforementioned *in vivo* findings by suggesting that an extranuclear milieu, i.e., the cytoplasm, of normal embryonic cells or eggs can override the nuclear genome in controlling the cellular phenotype. Therefore, leastways in these experimental settings, even if the nuclear genome bears oncogenic mutations, the mutations may not inevitably lead to neoplastic phenotypes. Many experiments have also shown that fusion of a cancer cell with a normal cell can make a phenotypically normal hybrid 415-424. However, mention should be made of that in many other occasions the hybrid cells may turn out to be more malignant 425. Actually, according to the reviews by Pawelek 426, 427 and Dittmar 428, already in the 1911 the pathologist Otto Aichel had proposed in a German language paper that fusion of tumor cells with leukocytes rendered the hybrid cells aneuploid and metastatic. Indeed, many studies have later shown that cell fusion may be a mechanism for tumor initiation and progression. It is worth mentioning that cell fusion is also a physiological event programmed in the mammalian genomes and occurring more often during embryonic stages 427-429. Since such hybrid cells contain both normal and neoplastic nuclei, whether and how this complicated system is related to embryonic milieu are unclear. The Parrondo's paradox, which says that losing strategies can work together to produce winning outcomes 425, 430, leads us to wonder whether the hybrid that doubles its number of chromosomes is fitter than the two original cells.

Mention should also be made of the plant evidence of the reversion, which has already been thoroughly reviewed by Braun in 1981 431. It has been shown, ever since 1926, that tumor cells in some plants can be reverted to normal plant cells and that tumor cells grafted onto another plant can develop into a normal plant which can bloom and produce seeds; the seeds can then germinate and grow to normal plants 432-453. What remains unknown is whether (or how) these observations on plants relate to the above-described data from embryonic environments of animals.

The above-described antithetic relationship between ePS or iPS cells and embryonic matrices, i.e. that stem cells in non-embryonic environment develop to tumors whereas embryonic environment reverts tumor cells back to phenotypical normal, extends the “seed and soil” theory that was initially proposed by Paget in 1889 to explain tumor metastasis 454: ePS or iPS cells as “the seeds” develop to normal tissues in one soil (embryonic environment) but to tumors in another soil (non-embryonic environment). Moreover, when the soil has changed (to an embryonic environment), the product of the seed (i.e. the tumor) may be changed (back to normal). These extended explanations of the theory favor the non-mutation theory as it is the environment (the “soil”), but not the cell (the “seed”) itself, decides whether the cell should develop into a tumor and, if it has already become a tumor, whether it should return back to normal again. The antithesis also dovetails with the initial “cancer stem cell” theory described by Julius Cohnheim in the 1870's 455, 456, which proposes that cancers are derived from stem cells in the normal tissues. Mention should be made of that there is a different “cancer stem cell” concept proffering that a tumor mass contains some cells that encompass properties of normal stem cells, such as self-renewal ability 138.

### Dissenting evidence 8: Immortality can be disengaged from transformation and other neoplastic properties in the lab

Some researchers have shown that cellular immortalization occurs before, and is a prerequisite of, neoplastic transformation 256-260, 457-461, which is the punditry of some other cancer wizards as well 258, 462-466. Ample animal studies have accentuated that tumor development undergoes a two-step procedure of initiation and promotion; in some peers' opinions, “initiation” immortalizes normal cells whereas “promotion” transforms the immortalized cells 467, 468. However, in 1983, Land et al showed that a mutant ras gene could transform embryonic fibroblasts *in vitro*, as these ras-expressing cells could form colonies in soft agar, but the transformed cells were still mortal because they could not grow indefinitely in the culture; their immortalization required concomitant expression of the c-myc or a viral oncogene 469. Similarly, mouse embryonic fibroblasts transformed with the SV40 large T antigen can efficiently form colonies in soft agar, but most of the cells will eventually die 470, 471. Concomitant expression of the CDK4 gene and a ras mutant can confer upon primary cells the ability to form colonies in agar and to develop into invasive tumors in animals, but the transformed cells remain mortal as evidenced by their limited passages in culture 472. These data suggest that *in vitro* neoplastic transformation can occur before, and thus can be extricated from, immortalization. Other studies have also shown this segregation 473, and there are data showing that simian virus 40 can transform human cells without immortalizing them 474. Telomerase alone has been shown to prod primary cells into growing in agar and in animals, which together is a well-accepted emblem of a neoplastic state, but these effects of telomerase are independent from immortalization 475-478 and transformation 467, 479. In some animal experiments, epithelial cells can be manipulated to invade, disseminate, and enter into the bloodstream before they can form primary tumors 480, 481; mammary epithelial cells can be manipulated to metastasize and colonize in the lungs before they are malignantly transformed 482, 483. All of the abovementioned laboratory data seem to suggest that immortality, transformation, invasion, and metastasis as key neoplastic properties can occur independently of one other and in any order, depending on the experimental setting. Because epigenetic alterations are reversible and occur more easily than mutations, it is much more easily fathomable if each of these key neoplastic features is caused by epigenetic alterations, and not by mutations, and thus can occur earlier or later than other neoplastic features. Of course, as aforesaid, other plausible explanations exist.

### Dissenting evidence 9: A neoplasm is a unicellular species and is somewhat genetically stable

As described earlier in this essay, the mutation theory says that cancer cells are genetically unstable and thus continuously accumulate mutations while endlessly replicating, leading to genetic heterogeneity 62, 63. It is therefore envisioned that over a long period of time each tumor lineage should have accumulated too many mutations that are too huge a burden for it to survive, meaning that no lineage of tumor, especially a very malignant one, can survive for a long time. However, a canine transmissible venereal tumor has survived for 11,000 years 484, and the Hela cervical cancer cell line has survived for seven decades 485; yet their genomes are still stable enough to maintain their lineages 484. The atavistic nature of carcinogenesis connotes that each cancer lineage is a new or semi-new species of unicellular organism 111, 112, 138. This “new species” concept denotes two important but often neglected notions: While “species” insinuates that a tumor lineage has a stable genome to forever maintain itself, “new” means that its genome has enough mutations to distinguish itself from its erstwhile one, i.e. the genome of its normal progenitor, because a species is defined by the specificity of the genome. It is possible that genomic instability of cancer cells, even at a chaotic extent, affects only certain parts of the genome while leaving certain other parts undamaged, and that once a mutant clone is selected, genomic stability resumes the hegemony until its cells enter into a new round of “mutations and clonal selections”, likely driven by new stress, and yield a newer mutant clone as a “newer species”. This is also to say that instability-caused mutations in cancer cells are not completely random and stochastic as they do not touch certain currently characterized core(s) of the genome that can later keep the “new” genome relatively intact. We envision that, if the Hela cell line was continuously cultured in dishes for another-thousand years, it would still be alive and be the Hela cells, although having millions of additional mutations. It seems that cancer researchers have emphasized enough the genomic instability of cancer but have put insufficient attention onto the aspect of their genomic stability. It remains unknown but very intriguing to us how a newly formed mutant clone, likely more malignant, turns from genomic instability to genomic stability.

## Cellular differentiation may be a mechanism for tumor reversion

In many (if not most) cases 306, 369-371, 376, 388, 389, 398, 486-491, reversion of cancer cells back to a normal state in an embryonic microenvironment occurs mechanistically via cellular differentiation 490, 492-497. With models of chick embryo and Zebrafish embryo, or with an intrauterine injection approach in mice, a slew of studies has shown that human malignant melanoma cells in an embryonic microenvironment do not develop to tumors but, instead, differentiate to neural-crest-like cells 498-501. Actually, earlier studies have shown that when the SRC oncogene is inactivated, the SRC-induced myosarcoma cells will differentiate into mature myocytes 502-504, and this inactivation-caused differentiation is actually a common event for SRC-caused transformation 505. Emphasis should be given to a study by Pierce and Wallace in 1971, in which some cells of squamous cell carcinomas were shown to differentiate into mature keratinized cells as squamous pearls 380. This observation is of significance as it shows that the squamous carcinoma cells highly resemble normal skin stem cells that divide asymmetrically to one stem cell (equivalent to a cancer stem cell) and one keratinocyte, and the latter continues both maturation and symmetrical division towards stratum corneum (equivalent to the other cancer cell that replicates and differentiates to the squamous pearl). Similar cellular differentiation has also been observed for the cells of chondrosarcoma as well as the cells of breast and colon cancers, which leads Pierce to conclude that the rules learned from teratocarcinoma govern the behavior of neoplasms in general 304, 306.

Certain extracellular matrices other than the embryonic milieu can also control cancer cells' phenotypes *in vivo*. The BAG2-GN6TF cells of rat hepatocyte origin may quickly develop into tumors or develop into normal hepatocytes in rats, depending on the sites and routes of the cell inoculation and on the age of the recipient rats 488, 506. S. Meryl Rose had also reported in 1948 that after frog kidney cancer cells were transplanted into and well grew in a salamander limb and then the limb was amputated through the tumor site, the limb could regenerate and some cancer cells within the regenerate differentiated into muscle and cartilage before they eventually died 507, 508. These earlier observations suggest that xenografted tumors can grow persistently in an alien animal species if the tumor cells remain undifferentiated, but if they differentiated to be more and more foreign, they will eventually be eliminated by the host. Shvemberger et al have in a series of publications reported that inoculation of mouse or rat malignant cells into an eye's anterior chamber of syngeneic animals can reduce the malignancy and increase differentiation of the tumor cells in association with a trend to normalizing the karyotype to diploidy, which presumably occur via selection of the subclones of cells that are relatively less malignant, more differentiated, and less aneuploid 90. However, these observations are partly incongruous with the seminal findings of Greene et al in the 1940's 138, 240. In a series of experiments, Greene et al. found that some cancers were transplantable to eyes' anterior chambers of those syngeneic animals that bear a tumor, but not of those without tumors 243, 509, 510. Greene et al also found that only those human tumors capable of metastasizing (but not those incapable) could be transplantable to eyes' anterior chambers of animals of a different species 509, 511-513. What remains vague is whether the various extracellular microenvironments described above are in a way related to an embryonic milieu.

Ever since almost a century ago 514-516, there have already been a battery of studies showing that certain extrinsic factors, such as some drugs or nucleic acids 385, 517-529, can facilitate the reversion of cancer cells back to normal via differentiation or maturation 89, 514-516, 530-532 in culture dishes, in animals, or in patients 468, 496, 533-536. Neural differentiation of the PC12 rat pheochromocytoma cell line induced by nerve growth factors or some chemicals is among the best-studied examples 537-539. A dietary supplement methylsulfonylmethane 540, which is also a normal oxidation product of dimethyl sulfoxide (DMSO) 541, can obviate metastatic properties of a few different cancer cell lines via differentiating the cells 542-546. Actually, there have been some clinical successes as proof in the remission of acute promyelocytic leukemias via differentiation induced by treatment with retinoic acid 547 or arsenic trioxide 548, 549, alone or in combination with other chemotherapeutic agents, although relapses from extant cells often ensue later 107. Some of these chemicals, with the arsenic trioxide being best studied, are known to effect via driving cells towards differentiation 548, 549. Cell lines from the abovementioned teratocarcinomas have been well studied for the molecular mechanisms of the reversion 550, 551. Other studies have suggested that reversion of leastways certain malignancies to a normal state may entail over 300 genes 378, 552-554.

Mention should be made of spontaneous regression of certain human cancers 90, 138, certain tumors in fish and amphibians 410, 555-563, and the canine transmissible venereal sarcoma 140, 564, 565. Reversion seen in some of these human and animal cases may in part be ascribable to differentiation and ensuing senescent death of the differentiated cells.

## Does mutation have anything to do with tumor reversion?

In Pierce's *apercu* 318, the above-described tumor reversion challenges the dictum of “once a cancer cell, always a cancer cell”. Simple explanations for the reversion include that the reversible tumors are not caused by mutations but by reversible epigenetic alterations 566 or, alternatively, that they are caused by mutations but the mutations are readily reversed back to normal 567. Actually, a “cell reversal theory” opines that carcinogenesis may start with reversal of a differentiated cell to a less differentiated epigenetic status, such as a stem cell status, whereas a stem cell or a cell at a stem status that does not dwell in the stem cell niche is very chaotic and will enter into uncontrolled proliferation 115. However, although all cancer researchers likely agree that epigenetic alterations are instrumental to the formation and progression of tumors 568, 569, whether or not such alterations alone are sufficient to cause tumors, especially the malignant ones, remains as an enchanting but fiendish puzzle. On the other hand, there are other equally plausible explanations for the tumor reversion, such as the three scenarios proposed by Telerman and Amson 378.

If tumors are caused by mutations as most cancer researchers believe, it seems improbable that the mutations would disappear later from live cells (lethal mutation may disappear along with the death of the cell 570). Therefore, a possibility is that the reverting pathway activated by the extrinsic reverting factors, such as an embryonic milieu or a chemical like retinoid acid or arsenic trioxide, is a different one from the mutations-initiated tumorigenic process and is not impeded by the mutations. Alternatively, the mutations may hinder the reversion but the extrinsic reverting factors can override the impediment, since correction of one or two signaling pathways has been shown to be capable of reverting cancer cells 496. In either scenario, the reverted cells are perceived to still retain the mutations 529, 571-574. In Harris' words, “the malignant phenotype may be held in an abeyance during the reversion” 418, which insinuates that the malignant phenotype can still reappear. Indeed, the animals developing from cancer-cell-derived gametes have a high chance to develop cancers late 575. Therefore, unless the normalized cells eventually die of senescence like all terminally differentiated cells 576, 577, thus purging mutations and cancer cells from the patient, the patient still faces a peril of tumor recurrence because, as aforementioned, the mutated genome still retains the right to control the phenotype. Today, with the feasibility of whole genome sequencing, repeating the early experiments described above and sequencing the whole genome of the cells before and after the reversion should help clarify these scenarios and provide us with information on what mutations the cells have that hinder the extrinsic-factor-driven differentiation of malignant cells.

## Our manipulations can only coerce primary cells into showing neoplastic features, but cannot directly transform the cells

We have previously realized a few attributes of experimental tumorigenesis 112, 138, 139, 578: 1) Lesions induced in most, if not all, animal models of tumorigenesis are inducer-dependent until terminal stages (for more early references, see 497, 579, 580). The lesions, even if they manifest cancerous morphology and behavior, regress upon withdrawal of the inducer, although reintroduction of the inducer usually 138, 581, 582, but not always 497, 581, 583, induces quick recurrence of the lesions. Regression of these non-neoplastic lesions occurs via apoptosis and thus differs from the aforementioned regression of tumors that occurs via differentiation and ensuing senescent death. 2) Cancer induction in animals requires a long latent time, and usually only one to several tumor masses appear in an animal 138, 276, 277. As aforesaid, these phenomena evince a negligible transformation efficacy of our manipulations at the cellular level. Besides these two properties, we have described three additional phenomena earlier in this essay: 1) Formation of tumors may not necessarily entail mutations. 2) Cells considered to be “transformed” may still be mortal. 3) Immortality, transformation, invasion, and metastasis as key neoplastic attributes can be segregated from one another in the lab and can occur in different orders, depending on the experimental setting.

**Figure 4:**
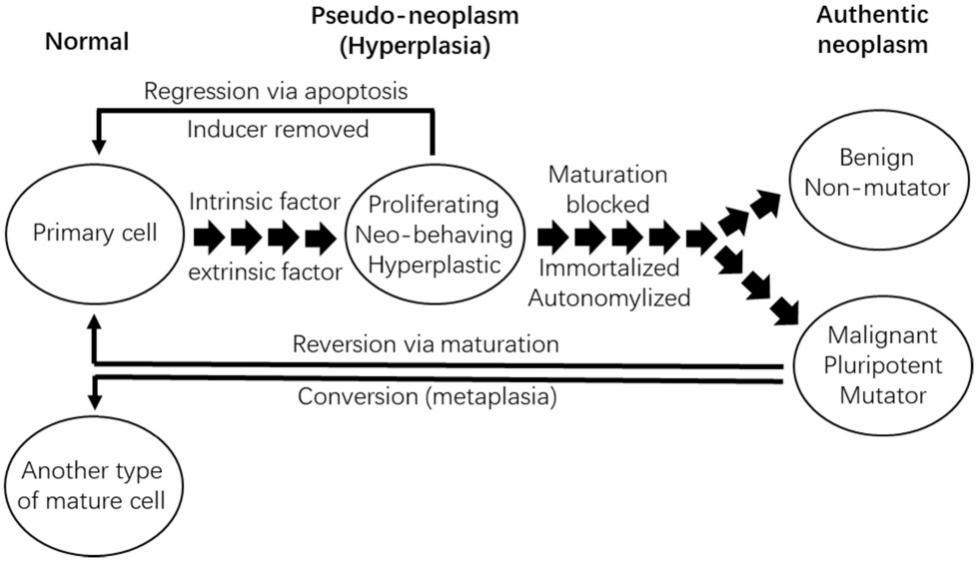
** Depiction of our coercion hypothesis and of tumor reversion or conversion.** Certain intrinsic factors (such as an inherited mutation) or extrinsic factors (such as our manipulation in an experimental animal) may drive proliferation of a primary cell to form an outgrowing lesion. If the factor disappears, such as due to the manipulator withdrawal, the lesion will regress via cellular apoptosis, suggesting that the lesion, which may have already exhibited cancerous features, has not yet become immortal and thus is still hyperplastic, and not yet neoplastic. At this stage, its proliferation and its possible manifestation of cancerous features are actually sustained under the coercion of the intrinsic or extrinsic factor. However, if the factor lasts much longer, the lesion will evolve to an authentic neoplasm by acquiring cellular immortality, autonomy, and maturation obstruction that are caused by epigenetic or genetic changes in non-mutator genes (in this case the neoplasm is benign) or in mutator genes (in this case it is malignant). The neoplasm, benign or malignant, may (or may not) be reverted back to normal like its normal counterpart tissue via cellular maturation, or may (or may not) be converted to another mature tissue type via metaplasia.

In our opinion, which is partly similar to Harris' punditry 416, all of the five traits of experimental tumorigenesis described above suggest that our manipulations in cell culture or in animals are not able to directly cause the cellular or molecular alteration(s) that bestow immortality and autonomy upon the primary cells. In most, if not all, of our *in vitro* or *in vivo* systems, our manipulation, such as knockout of the p53 gene or ectopic expression of a k-ras mutant, is simply to coerce the primary cells into 1) replicating incessantly, 2) manifesting transformed morphology and/or behavior, 3) sustaining the cells' life, 4) causing or accelerating DNA damage, and 5) impairing DNA repair mechanisms 112, 138, 139, 241, 584. The lesions produced are actually hyperplastic, and not neoplastic. Actually, the malignant behavior of these hyperplastic cells had already been observed in the world's first experiment of chemical tumorigenesis by Fischer in 1906 585. According to Braun 431, Fischer repeatedly injected Scharlach R into subcutaneous sites of rabbits' ears, which drove the local epithelial cells to proliferate and invade deeply into the blood and lymphatic vessels. In some animals the lesions metastasized distantly. However, the cells, although invasive and even metastatic, remained mortal as they regressed upon withdrawal of the Scharlach R. The cellular alterations directly responsible for the immortality and autonomy, which are still unknown to us even now (Fig. 4), can only occur spontaneously in a random and stochastic manner during the incessant cell replication under the duress from our manipulations. This is why when the transforming agents, such as oncoviruses, are withdrawn or lost, the “transformed” cells may revert back to normal, a phenomenon that has already been discerned for over 50 years 586-588 and reviewed many years ago 497. Actually, sometimes our manipulations can just confer upon primary cells additional rounds of cell replication, as epitomized by additional 20-30 population doublings of primary cells offered by ectopic expression of the SV40 large T antigen, during which a few cells acquire spontaneous cellular or molecular alterations that establish immortalization 589. Pierce had once stated in 1983 306: “it is easy to show what cells can be made to do, and it is often difficult to know what cells do.” We should remind ourselves that what we have observed in our experiments is what cells are forced by us to do, but what we actually want to know is what cells, and even the organism (such as a human being) as a whole, would like to do in a given physiological or pathological situation 6, 68, 112, 138, 578, 584. Probably, we often put the cart before the horse in our research 112.

Our manipulations drive cell proliferation to form hyperplastic lesions, cells of which are redundant and still allegiant to the animal's body. This allegiance forces the cells to commit suicidal apoptosis and probably, to a lesser extent, also senescent death, because the animal's body wants to avoid cellular redundancy of the tissue or organ 111, 138, 139, 241, 546, 576, 590-595. It is likely that our manipulations inhibit apoptosis and senescent death as components of their coercive mechanisms, but this inhibition disappears once our manipulations are withdrawn. Oncogene-withdrawal-caused regression of the-oncogene-induced outgrowths may involve modification of metabolism 596 and immune functions 597, 598, which is not surprising as the cells die via apoptosis and apoptotic cells are known to be eliminated via phagocytosis by macrophages, according to Kerr et al who created the word “apoptosis” 599. Since in culture systems cells do not have to care about the cellular redundancy issue, a spellbinding but unaddressed question is whether, after withdrawal of the coercion, the proliferating cells die of senescent death or/and some other form(s) of programmed cell death 138, 577.

Knowing that there is no way of promptly immortalizing primary cells, researchers often perpetuate the manipulations, namely the coercions, by using such as stably-expressing cell clones or transgenic animals. However, for different research needs, many systems of “conditional immortality” or “conditional transformation” have also been created 600-605, including transgenic animals 606. Accordingly, many conditional cell lines have been established 589, 606-615, like the temperature-controlled ones 612, 616, which show controllable immortalization or neoplastic transformation 612, 615, 617. The words “conditional” and “controllable” already proclaim the nature of swift reversibility and accentuate that the immortality or the neoplastic transformation so created is not authentic because the cells are still mortal.

Bearing the manipulation-bestowed duress in mind, many “surprising findings” in animal models are actually not so surprising, such as the aforementioned observations that epithelial cells can evade, disseminate, and enter into the bloodstream before they can form primary tumors 480, that cancer cells can enter into the circulation before invading adjacent stroma 481, and that mammary epithelial cells can metastasize and colonize in the lungs before they are malignantly transformed 482, 483. These results from manipulated animals show diversion from the “growth, invasion, and then metastasis” trajectory of epithelial carcinogenesis 62, 295. These phenomena have not and will not be discerned in human situations, because withdrawal of the coercers will likely lead to the disappearance of these comportments of manipulated cells.

Most, if not all, of our manipulations in experimental systems of tumorigenesis have been designed to simulate epigenetic or genetic alterations identified in human tumors. For instance, we often ectopically express a k-ras mutant in pancreatic ductal cells to transform them because we know that most pancreatic cancers bear this mutation 251, 618, 619. However, we need to bear several points in mind: 1) In human tumors, these alterations are not the intrinsic factors directly responsible for the tumor cells' immortality and autonomy, although they might have already caused, by kindling a cascade of molecular events, cellular immortality and autonomy at the time of diagnosis. 2) In many, if not most, experimental studies, the target cells may not have been immortalized but have already displayed transformed comportments and/or morphology, which may dupe us into discontinuing our manipulations and harvesting the lesions before they have experienced spontaneous immortalization and become genuine neoplasms. 3) The molecular or cellular aberrations we conferred onto primary cells, such as k-ras mutations, can transform the cells in culture dishes and in animals, but it does not mean that there actually is a patient whose tumor is caused by one of these anomalies. There are probably over 100 million genetic alterations of different types in human cancers, since there have been about 85 million point mutations identified 620, 621, pancreatic cancer alone has 857,971 genetic alterations identified 622, and the p53 gene alone has over 30,000 mutation types 6, 623. A fact is that many cancer researchers endlessly use different combinations or different sequences of these alterations to efficiently transform primary cells in culture or to precipitate tumors in animals, and then claim identification of novel carcinogenic pathways. However, researchers are still unable to pinpoint any of these alterations, these combinations of alterations, or these orders of alterations, which together make innumerable permutations, as the cause for the tumor formation in a patient 112. Even worse, it remains possible that these alterations, or these combinations or sequences of alterations, are just the results or byproducts, but not the causes, of the tumor formation in patients.

## Only the neoplastic morphology and behavior caused by intrinsic factors are authentic

**Figure 5:**
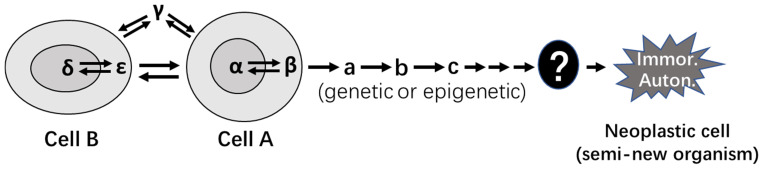
** Illustration of the relationship between the “tumor-initiating factors” and their downstream “immediate tumor-causing factors”.** A cell (cell A) may have an epigenetic or genetic alteration in the nucleus (α) that occurred spontaneously, was inherited from a parent, or was caused by an altered factor in the cytoplasm (β) or by an extracellular factor (γ, such as a radiation, a chemical, or a virus). A similar alteration in the nucleus (δ) or cytoplasm (ε) may also occur in another cell (cell B) nearby or even in a distant body site, which alters the communications and interactions with cell A, in turn causing α or β. All of these alterations may mutually affect each other (between the two cells, between the nucleus and the cytoplasm of a cell, as well as between the intracellular and extracellular environments of a cell). α or β is defined herein as a “tumor-initiating factor” as it triggers a cascade (referred to as a, b, c, etc.) of molecular events in the nucleus (e.g., epigenetic or genetic changes) and/or the cytoplasm, culminating in one or some currently-unknown cellular or molecular alterations (question mark) that establish cellular immortality (Immor.) and autonomy (Auton.), namely a neoplastic state, and thus are coined herein as “immediate-tumor-causing factors”.

The above-described “coercion hypothesis” signifies an important fact learned from over a century of experimental tumorigenesis research: *In vitro* colony formation, neoplastic morphologies, as well as invasive and metastatic behaviors can all be caused by both extrinsic and intrinsic factors. The currently-unidentified cellular or molecular alterations responsible for immortality and autonomy are intrinsic factors, and the neoplastic morphology and behavior caused by them reflect an authentically neoplastic state (Fig. 5). On the contrary, those neoplastic morphology and behaviors occurring under the duress from our manipulations, which are extrinsic factors, do not reflect a neoplastic state.

The notion that only intrinsic-factor-caused neoplastic properties are authentic repudiates extrinsic-factor-caused spuriousness, and thus is of importance and has clinical relevance. Many things, such as chronic viral or bacterial infections, treatments with certain drugs, exposures to certain environmental pollutants, etc., may be such extrinsic factors that coerce cells into outgrowing and manifesting neoplastic features. For example, chronic infection by Helicobacter pylori (HP) can result in low-grade lymphomas 624-630, chronic infection by human T cell lymphotropic virus type I (HTLV-1) can cause lymphoma or leukemia 631-633, and infection by parasite theileria can transform bovine leukocytes into disseminating tumors 634-636. However, therapeutic removal of these causal pathogens can cure these tumors, leastways at an early stage. For another example, hepatomas and hepatocellular carcinomas had been reported frequently during the 1970's-1980's among women chronically using estrogen-rich oral contraceptives, but the tumors could regress upon cessation of the contraceptives 637-643. In these instructive cases, the cure of the tumors upon removal of the extrinsic factors is reminiscent of the withdrawal of our manipulations in experimental systems. In our opinion, the tumor cells caused by the HP, HTLV-1, theileria parasite, or excessive estrogen may not have been immortal and autonomous at the time of diagnosis and thus may not be authentically neoplastic, albeit their morphology denotes a pathological diagnosis of malignancy and they, if left untreated, will eventually evolve to genuine neoplasms.

## We still have no way of directly transforming primary cells

In all experimental systems established so far, our manipulation can make primary cells of small rodent origins truly immortal only after weeks in cell culture or months in animals 138, obviously as a secondary event. Some plant cells may be exceptions, as some early studies showed that some plant cells could be transformed after only 34-48 hours of manipulation 440, 443, 453, with a few more days of manipulation creating more aggressive cells 431, 441, 442, 444-446, 449-451. This is to say that in our *in vitro* and *in vivo* models, the cellular alterations responsible for immortality and autonomy occur only spontaneously during the enduring cell replication caused by the duress from our manipulations (Fig. 5). The aforementioned fact that usually only one to several of the cells in an animal develops into tumors signifies that normal cells guard firmly their mortality program to ensure that all cells will die eventually, which is the will of a higher eco-system as we expounded before 241. We still hitherto have had no way of breaking through this guard of normal cells and thus have to wait until the cells themselves give up this guard to adapt to the stressful milieu. Fortunately, our manipulations as extrinsic factors can accelerate this giving-up not only by imposing a stressful environment but also by sustaining the cells' life, accelerating cell replication, damaging DNA, impairing DNA repair, etc.

## There are two types of differentiation obstructed by two types of cellular alterations

All neoplastic cells, benign or malignant, are less differentiated than their normal counterparts, as pointed out decades ago by Markert 288, betokening that cellular maturation has been blocked during tumorigenesis 644, 645. Of course, in benign tumor cells this blockade may be set at a point near the terminal differentiation, allowing the cells to highly resemble their normal counterparts. For example, uterine leiomyoma cells are highly similar to, and thus basically indistinguishable from, uterine muscle cells in cellular morphology; these tumors are pathologically diagnosed mainly based on their histological features.

There are three different sets of antithetical cellular properties that pertain to the obstruction of maturation (Table 1). First, a normal cell can be well-differentiated but still possesses a strong proliferation potential 646, 647, evincing that differentiation and proliferation are not incompatible 44, 149, 415, although cells that proliferate robustly are usually less differentiated. As noted by Harris 415, 648, it has become an “ancient question of whether a tumor grows rapidly because it does not differentiate or does not differentiate because it grows rapidly, but this is a false question.” For instance, after partial hepatectomy, the remaining hepatocytes that are highly differentiated can robustly proliferate to produce new well-differentiated hepatocytes 6, 649. Second, immortality, which can be considered as an extreme of proliferation potential, and differentiation are not incompatible either. For instance, many benign tumor cells not only are immortal but also are well-differentiated, with uterine leiomyoma cells as an epitome. Third, even very malignant cells from the same patient can differentiate into a diversity of tissue types 650-658, which is a phenomenon often dubbed as “metaplasia” or “transdifferentiation” 385, as in pathology textbooks “metaplasia” means conversion from one differentiated cell type to another, such as squamous metaplasia and osseous metaplasia. Actually, benign tumor cells from a given patient may exhibit multiple types of metaplasia as well 659-661. This betokens that tumor cells may retain pluripotency, although they are blocked somewhere towards the terminal maturation. Therefore, there are two different types of cellular differentiation, one being maturation towards the parental cell or tissue type, and the other being metaplasia towards some other cell or tissue type(s). This fact further annunciates that the currently-unidentified molecular or cellular alterations which militate against cellular differentiation can be dichotomized into two categories, i.e., 1) those that prevent tumor cells from maturation without stymieing their pluripotency and thus allowing the cells to differentiate into one or more other cell types, and 2) those that not only interdict maturation but also cancel pluripotency. A captivating question oblivious of by many researchers is whether immortality, autonomy, and maturation interdiction are three different facets of the same dice, i.e., whether these three neoplastic properties are controlled by the same cellular factor(s).

One important concept learned from the above introduction is the existence of three cellular antitheses, i.e., 1) well-differentiated status vs proliferation potential of normal cells, 2) immortal status vs well-differentiated status of benign tumor cells, and 3) maturation blockade vs pluripotency, or maturation disability vs metaplasia ability (Table 1). Another important notion is that, germane to tumorigenesis, one type of cellular or molecular aberration is those stymieing only cellular maturation and another type is those impeding both maturation and pluripotency. Being cognizant of these two concepts is of importance, because we may consider developing some approaches or extrinsic factors as remedies for directing cancer cells towards certain types of metaplasia as an alternative, if it is difficult or impossible to direct the cells towards maturity such as in the situation where maturation genes are severely impaired 467. Either type of differentiation should be followed by senescent death of the cells 576, 577.

## Immortality and autonomy may entail one set of genes, while malignant morphology may involve another set

A prodigious number of publications deliver, to many cancer biologists and molecular biologists who lack clinical experience in oncology and surgical pathology, a convoluted message about the demarcation between benign and malignant neoplasms. For instance, most of the “cancer hallmarks” described by Hanahan and Weinberg 662, 663 are actually not unique to malignancy, and certainly are not unique to every cancer cell in the same cancer mass. They are, in fact, hallmarks of “any growing tissue”, in Llambi's words 664, including benign neoplasms, as pointed out first by Lazebnik 665 and later by us 112. In Blagosklonny's words, “…hallmarks can be observed without cancer” 475. Today, there have not been any molecular markers available for us to distinguish malignity from benignity, and morphological features are still the main clinical criteria for this differentiation. However, morphological criteria are not flawless, as has been pointed out by the superlative surgical pathologist Harry S. N. Greene in 1948 509 and has been reviewed by us 138. Concerns about pathological criteria include overdiagnosis 666, 667, such as overdiagnosis of thyroid cancer 668-671. Therefore, we may need to find a better way to classify tumors or to reset the demarcation between malignity and benignity so as to better explain a tumor's prognosis.

Both benign and malignant cells are immortal and autonomous. However, many benign cells highly resemble, whereas most malignant cells differ greatly from, their normal counterparts in morphology. This disparity connotes that there are some epigenetic or genetic alterations establishing only immortality and autonomy without significantly affecting cellular morphology, whereas there are some other alterations that specifically establish malignant morphology and do not occur in benign tumors. We surmise that there may be a set of genes, or one or more genomic structures depicted in Figure 1, that govern not only cellular mortality but also the loyalty of cells to their host body; their alterations, epigenetic or genetic, establish cellular immortality and autonomy. We herein call these genes or genomic structures “mortal and loyal factors” and call their alterations “immediate tumor-causing factors”, so as to distinguish them from well-studied oncogenes or tumor suppressor genes that have well-known roles in initiating a lengthy tumorigenesis (Table 2 and Fig. 5). Conversely, there may be another set of “malignant morphology genes or genomic structures”, dubbed herein as “malignant morphology factors” for simplicity, whose anomalies are responsible only for the establishment of malignant morphology (Table 2). An enthralling but unaddressed question is whether the “malignant morphology factors” are also those controlling cellular maturation, since maturation pertains not only to morphology but also to function.

So far, there has not been any “mortal and loyal factor” or “malignant morphology factor” established, although whole genome sequencing has been performed on thousands of tumors. Probably, as Heng et al has frequently pointed out before 39, 41, 42, 672, one of the reasons is that these factors or some of them are not genes shown as the level 1a in Figure 1 but entail higher genetic levels. Moreover, in our opinion, one tactical mistake cancer researchers have made for many decades is to dwell mainly in the research of malignancy and hardly set foot onto research of very benign neoplasms. Very benign neoplasms, typified by uterine leiomyoma, are likely to have many fewer and much stabler genetic and epigenetic alterations, compared to their malignant counterparts. Therefore, they serve as much simpler and thus better models for us to identify the critical alterations immediately behind cellular immortalization, autonomization, and probably also maturation interdiction.

The benignity or malignity, and even the neoplastic state, of cells transformed *in vitro* require much more attention. Some cell lines such as MCF10AT 673, 674 can form colonies in soft agar, which is considered an insignia of a neoplastically transformed state 251, 675, but in animals they can form only benign tumors, judged by the histology of the xenograft tumors. On the other hand, some other cell lines, like NMuMG (ATCC website, 676, and our experience), cannot efficiently form colonies in agar but can often form benign tumors in animals. These and other dissonant lab data lead us to consider that both colony formation in culture and xenograft tumor formation in animals are required to qualify a neoplastic transformation. Determining whether *in vitro* transformed cells are malignant is difficult, and currently we still lack convenient but reliable measures for this purpose 251, since the soft agar clonogenic assay initially developed by Hamburger and Salmon in 1977 677 is not always reliable 678, 679. Cell fusion studies have suggested that transformed and malignant phenotypes are under separate genetic control 680, which is fathomable because a transformed, i.e., neoplastic, state may be benign. As Lazebnik has pointed out, distant metastasis is currently the only reliable yardstick for malignancy 665, although this canon is still not flawless because in some rare cases histologically benign lesions also metastasize, such as in the cutaneous fibrous histiocytoma 681 and in the growing teratoma syndrome of the ovary 682, 683. Unfortunately, most relevant studies employ only subcutaneous inoculation of *in vitro* transformed cells, whereas few cell lines at a subcutaneous site can metastasize distantly, according to the literature and our experience, although we suspect that some cell lines may metastasize if inoculated viscerally.

## Benignity and malignity may be defined based on genomic alteration

Benign tumors in general do not progress but malignant ones are always on their way to more-wayward states, notably states of metastasis and therapeutic resistance. Tumor progression is perceived to be attributed chiefly to accumulation of more epigenetic or genetic alterations, which is in turn ascribed to certain initial alterations, such as those impairing DNA repair. Pertinent to progression, tumor-related genes can be dichotomized into 1) mutators that are defined herein as the genes or genomic structures whose epigenetic or genetic alterations can cause or accelerate alterations at others and 2) non-mutators whose epigenetic or genetic alterations do not cause alterations at others (Table 2). While the “evolvability” theory of Pienta et al suggests the involvement of the ability of evolution in tumorigenesis 141, 684, our “mutator” concept, which may entail any genomic level(s) illustrated in Figure 1 and not just canonically defined genes, emphasizes the ability of evolution in tumor progression to more-heinous states. With this dichotomy, neoplasms can be reclassified at the genomic level: Benign neoplasms are those bearing epigenetic or genetic anomalies at non-mutators and thus do not accumulate genetic abnormalities, whereas malignant neoplasms are those bearing epigenetic or genetic alteration(s) at the mutators and thus easily have accrued alterations (Table 3 and Fig. 4) as the bedrock for continuous progression towards more-diabolical states 62. The essence of this reclassification is first to attribute accumulation of epigenetic or genetic alterations to the initial ones at certain mutator(s), then to attribute progression potential to the accrual of such alterations, and finally to utilize progression potential to demarcate the border between benignity and malignity. Of course, benign cells are also immortal and keep replicating, which increases the risk for new alterations to occur. Actually, this is a reason why some benign tumors are at peril for progression.

**Figure 6:**
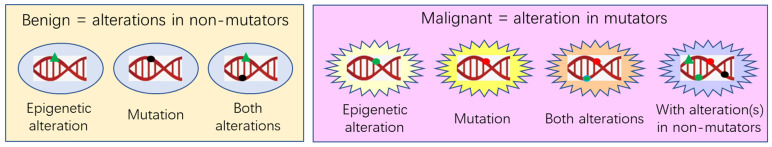
** Propounded classification of tumors.** Benign tumors are those bearing epigenetic alterations (green triangle) and/or mutations (black dot) in non-mutator genes or genomic structures. Malignant tumors are those bearing epigenetic alterations (red dot), mutations (green dot), or both in mutators, with or without epigenetic alterations and/or mutations in non-mutators.

Epigenetic aberrations more often change the expression level of the inflicted gene than confer new function onto it, whereas mutations may completely change the gene's function. Therefore, epigenetic changes may or may not resemble mutations. It is perceivable that epigenetic alterations of mutators may trigger epigenetic and genetic changes at other mutators and non-mutators and thus may drive tumor progression as well. With our reclassification, benign tumors can be further systemized into 1) those bearing epigenetic alterations only at non-mutators, 2) those bearing mutations only at non-mutators, and 3) those bearing both. Similarly, malignant tumors can be further stratified into several subgroups as those with or without epigenetic or genetic changes at non-mutators, besides the alterations in mutators (Table 3 and Fig. 6).

Currently, our classification approach can only be used in the study of neoplastic comportments and underlying mechanisms, and is inapplicable in clinical practice because we still do not have enough detail about which genes or genomic structures are mutators and what alterations they have that are implicated in establishing malignancy, although many mutations of many genes are already considered by some cancer pundits as cancer “drivers” 62, 685-689. However, as aforesaid, our classification provides an explanation for the question as to why some rare tumors can regress spontaneously or can be cured easily: They may bear only alterations in non-mutators without involvement of epigenetic or genetic alterations in mutators, and are benign even if they manifest malignant morphology. Moreover, there is an obvious incongruity between morphology and prognosis seen now and then in the clinics. For example, most nasopharyngeal cancer**s** are the undifferentiated type, but many of them actually have a good prognosis and can even be cured 690-692, which sharply contrasts with most other types of poorly differentiated or undifferentiated cancer that have very poor prognoses. It is captivating to know whether those undifferentiated but curable cancers bear alterations only in non-mutators.

## Above-described mouse teratocarcinomas may be special with little human relevance

In clinics, teratoma and teratocarcinoma are usually pediatric pathologies, although teratoma in some males may be diagnosed as late as middle age 693. Pediatric tumorigenesis has its inception at an embryonic stage 694 and may indeed occur as a repercussion of epigenetic aberration. In our rumination, teratomas occur simply because epigenetic or genetic changes occur to some early pluripotent cells and thwart their differentiation while the cells proliferate continuously, whereas teratocarcinomas occur because such alterations occur at an even earlier embryonic stage and the hindrance of differentiation makes the tumor cells less differentiated. Reiterated, if less-differentiated pluripotent cells are the tumor progenitors, teratocarcinomas would result, whereas if more-differentiated cells are the tumor progenitors, teratomas would result 318. However, even if epigenetic alterations were the initial causes, in real life these tumors have likely developed mutations as secondary events and become irrevocable at the time of diagnosis. If other types of pediatric neoplasms are initiated by epigenetic aberrations alone as well, many of them may have also acquired some mutations at the time of diagnosis, or even before the child was born. The 40-week gestation is a long stint during which a single fertilized egg grows into a fetus of several kilograms, involving numerous rounds of cell replication and thus providing numerous opportunities for mutations to occur. Actually, if certain rare sporadic tumors in adults are also initiated by epigenetic alterations alone, the tumors have likely developed mutations at the time of diagnosis as well. Therefore, in real life the adult cancers that bear only epigenetic aberrations are probably as scarce as hen's teeth. We realize that there are some tumors without mutations detected, but several possibilities remain to be ruled out. First, some single nucleotide polymorphisms in these tumors may actually function as mutations. Second, mutations on the extrachromosomal DNA 695, 696 or alterations at the level(s) of genomic structures higher than the gene level (Fig. 1) have been neglected or are harder to discover. Third, technical issues may exist 620, 697, 698. Actually, we still do not know what mutations are responsible for immortality and autonomy and thus do not know what we should specifically look for. However, tumors in small rodents may show epigenetic alterations alone, partly due to their much shorter lifespans and smaller body sizes, besides other disparities from humans 699. Because cancer cells in the human and the mouse require similar time frames for completing one cell cycle, which is around 24 hours for those fast-proliferating cell lines according to the literature and our experience 6, a tumor of the same size in mice and in humans has a similar cell number, but a very small tumor in a human is already very large in a mouse. Therefore, tumors in mice are much smaller and have experienced far fewer rounds of cell replication, thus having far fewer chances to develop mutations, generally speaking. In reality there is no way of knowing whether or not clinically-diagnosed human cancers are solely caused by or bear only epigenetic alteration(s).

## Concluding remarks

There have been many theories about tumorigenesis, disputing over the involvement of mutations. One extreme theory considers that mutations not only are the initial cause but also reach a chaotic extent, while the diametrical theory thinks that mutations are not necessary. Main evidence against the mutation theory includes that ePS or iPS cells displaced in a non-embryonic environment may develop to neoplasms, whereas neoplastic cells placed in an embryonic environment may be reverted back to phenotypic normal. Until now, the differential control by the embryonic environment in this antithesis still remains vague. In our opinion, both extremes and many, if not all, intermediate theories are correct as they describe formations of different types of neoplasms in different situations. We envision that a chaotic level of genomic changes can occur in highly stressful situations and can more efficiently establish a malignant state. For example, isolating a primary cell and putting it into a culture dish containing a medium with 10% fetal (but not adult) bovine serum make the cell highly stressed, because it is nourished abnormally and has lost all interactions with other cell types and lost normal neural and hormonal controls. Forcing the cell to ectopically express one or more oncogenes, which is a common approach to transform cells, further raises the stress level. However, it remains questionable whether in patients the genome still has to experience a chaotic mess for development of some benign tumors, such as uterine leiomyoma that is indistinguishable from normal uterine muscle in most cellular aspects and may be caused by mild hormonal imbalance. Theoretically, certain epigenetic changes in nuclear proteins, such as abnormal phosphorylations of histones, may alter some genomic structures such as nucleosomes and chromosomes, in turn initiating tumor formation. While carcinogenesis has been extensively studied, an important aspect of tumorigenesis, i.e. development of benign tumors in a slightly abnormal situation, has been much understudied. In turn, fewer discourses have been focused on the immediate tumor-causing factors, i.e., those molecular or cellular alterations that directly establish cellular immortality and autonomy. The “immortality and autonomy” definition of neoplasia connotes that a neoplasm resembles a new or quasi-new unicellular organism 700 and thus should have some mutations, because a new organism should have something new in the genome 141. Therefore, wrangling over “whether epigenetic abnormality alone can establish cellular immortality and autonomy, namely establishing a neoplastic state”, is actually a debate on whether “difference(s) only at the epigenetic level are sufficient to define a new organism”, making this issue a general question of taxonomy. In our opinion, neoplasms are malignant if they bear epigenetic or genetic abnormalities in mutator genes or genomic structures, defined as those whose alterations accelerate others to change, whereas neoplasms bearing epigenetic or genetic abnormalities only in non-mutators are benign. Future mechanistic research should be devoted to identifying the abovementioned “immediate tumor-causing factors”. Very benign tumors may have many fewer alterations and thus be much simpler and better models than malignant ones for this line of research 701, 702. Future therapeutic research should be focused on identifying the extracellular and intracellular factors (such as embryonic ones) that control tumor cells' phenotypes and on establishing approaches or drugs that can revert cancer cells to a differentiated state, either maturation or metaplasia.

## Figures and Tables

**Table 1:** Three sets of opposing cellular properties relevant to neoplasms

Cell type	Maturity	Opposing properties
Normal	Mature	Proliferating and differentiated
Benign	Blocked at late differentiation stage	Immortal (endlessly proliferating) and differentiated
Malignant	Blocked at early stage	Undifferentiated and pluri-differentiating potency

**Table 2:** Classification of genetic factors relevant to key properties of tumor biology

Category	Features/Functions	Current state	
Tumor-initiating factors	Oncogenes	Well studied	
	Tumor suppressor genes		
Mortal and loyal factors	Block maturation and establish immortality and autonomy	Hypothetical; unidentified	

Tumor morphology factors	Benign (similar to normal)	Hypothetical; unidentified	
	Malignant (greatly divergent from normal)		
Tumor progression factors	Non-mutators related to benign tumors	Some identified as oncogenes or tumor suppressor genes	
	Mutators related to malignant tumors		

Note: The “genetic factors" may be canonically defined genes on the genomic DNA sequences but can also be higher genomic structures depicted in Figure 1.

**Table 3:** Tumor classification at the genomic level

Type	Non-mutator		Mutator		Properties
	Epigenetic	Mutation	Epigenetic	Mutation	
I	with	without	without	without	benign, easily cure
II	without	with	without	without	benign, curable
III	with	with	without	without	benign, curable
IV	with/without	with/without	with	without	malignant, relatively better
V	with/without	with/without	without	with	malignant, bad
VI	with/without	with/without	with	with	malignant, worse

Note: mutator and non-mutator include not only canonically defined genes but also higher structural level(s) shown in Figure 1.

## Abbreviations

DMSO: dimethyl sulfoxide
ePS or iPS: embryonic or induced pluripotent stem (cells)
HP: Helicobacter pylori
HTLV-1: human T cell lymphotropic virus type I
TOFT: tissue organization field theory
3T3: transferring 3×10^3^ cells from one flask to another every 3 days
